# Identification of Ferroptosis-Related Genes Signature Predicting the Efficiency of Invasion and Metastasis Ability in Colon Adenocarcinoma

**DOI:** 10.3389/fcell.2021.815104

**Published:** 2022-01-26

**Authors:** Chunlei Shi, Yongjie Xie, Xueyang Li, Guangming Li, Weishuai Liu, Wenju Pei, Jing Liu, Xiaozhou Yu, Tong Liu

**Affiliations:** ^1^ Department of General Surgery, Tianjin Medical University General Hospital, Tianjin, China; ^2^ Tianjin General Surgery Institute, Tianjin, China; ^3^ Key Laboratory of Cancer Prevention, Department of Pancreatic Cancer, National Clinical Research Center for Cancer, Tianjin’s Clinical Research Center for Cancer, Tianjin Medical University Cancer Institute and Hospital, Tianjin, China; ^4^ Department of Breast Oncoplastic Surgery, Tianjin Medical University Cancer Institute and Hospital, National Clinical Research Center for Cancer, Key Laboratory of Cancer Prevention and Therapy, Tianjin's Clinical Research Center for Cancer, Key Laboratory of Breast Cancer Prevention and Therapy, Tianjin Medical University, Ministry of Education, Tianjin, China; ^5^ Department of Molecular Imaging and Nuclear Medicine, National Clinical Research Center for Cancer, Tianjin Medical University Cancer Institute and Hospital, Tianjin, China; ^6^ Department of Pain Relief, Tianjin Medical University Cancer Institute and Hospital, National Clinical Research Center for Cancer, Key Laboratory of Cancer Prevention and Therapy, Tianjin, China

**Keywords:** COAD, metastasis, ferroptosis, risk model, WGCNA, PCOLCE, HOXC11

## Abstract

**Background:** Colon adenocarcinoma (COAD) is one of the most prevalent cancers worldwide and has become a leading cause of cancer death. Although many potential biomarkers of COAD have been screened with the bioinformatics method, it is necessary to explore novel markers for the diagnosis and appropriate individual treatments for COAD patients due to the high heterogeneity of this disease. Epithelial-to-mesenchymal transition (EMT)-mediated tumor metastasis suggests poor prognosis of cancers. Ferroptosis is involved in tumor development. EMT signaling can increase the cellular sensitivity to ferroptosis in tumors. The aim of our study is finding novel prognostic biomarkers to determine COAD patients for predicting efficiency of metastasis status and targeting precise ferroptosis-related therapy.

**Methods:** A novel gene signature related to metastasis and ferroptosis was identified combing with risk model and WGCNA analysis with R software. The biological functions and predictive ability of the signature in COAD were explored through bioinformatics analysis.

**Results:** We established a four-gene prognostic signature (MMP7, YAP1, PCOLCE, and HOXC11) based on EMT and ferroptosis related genes and validated the reliability and effectiveness of this model in COAD. This four-gene prognostic signature was closely connected with metastasis and ferroptosis sensitivity of COAD. Moreover, WGCNA analysis further confirmed the correlation between PCOLCE, HOXC11, and liver and lymphatic invasion of COAD.

**Conclusion:** The four genes may become potential prognostic biomarkers to identify COAD patients with metastasis. Moreover, this four-gene signature may be able to determine the COAD suitable with ferroptosis induction therapy. Finally, PCOLCE2 and HOXC11 were selected individually because of their novelties and precise prediction ability. Overall, this signature provided novel possibilities for better prognostic evaluation of COAD patients and may be of great guiding significance for individualized treatment and clinical decision.

## Introduction

Colon adenocarcinoma (COAD), one of the most prevalent cancers worldwide, has become a leading cause of cancer death (Bray et al., 2018; Miller et al., 2019). Over 1 million new COAD cases and 551,269 deaths occurred in 2018, accounting for about 1 in 20 cancer cases and deaths (Bray et al., 2018). It is estimated that more than 2.2 million new cases and 1.1 million colorectal cancer deaths occur by 2030 (Arnold et al., 2017). Despite the improvements in surgical and medical therapies, the 5-years survival rate of COAD patients is still unsatisfied in the past several decades (Kuipers et al., 2015; Siegel et al., 2020). Moreover, the 5-years survival rates for patients with early stage cancer are approximately 90%, whereas for patients diagnosed with advanced-stage metastatic disease is only 10% (Kuipers et al., 2015), indicating that metastasis is a critical cause of death for cancer patients. Therefore, there is an urgent need to identify novel prognostic biomarkers related to the tumor metastasis, which may facilitate the timely diagnosis and application of appropriate individual treatments for COAD patients.

At this time, numerous studies have focused on screening new genes and established multi-gene prognostic models. Zuo et al. identified a 6-gene signature (EPHA6, TIMP1, IRX6, ART5, HIST3H2BB, and FOXD1) significantly associated with the survival of colorectal cancer patients (Zuo et al., 2019). Liu et al. constructed an invasion-related 6-gene signature (ITLN1, HOXD9, TSPAN11, GPRC5B, TIMP1, and CXCL13) that could evaluate the prognostic risk of patients with colon adenocarcinoma (Liu et al., 2021). Moreover, Wang et al. established a 12-gene based prognostic signature based on DNA damage and repair related gene sets that is beneficial for the evaluation of clinical prognosis on colorectal cancer patients (Wang X.-Q. et al., 2021). Further data mining using the public datasets *via* different angles may display other prognostic-related genes and construct a reliable prognostic model, which may provide a tool for prediction of prognosis and selection of individualized treatment.

Invasion and migration of cancer cells into peritumoral tissues and organs could result in cancer metastasis. Epithelial-to-mesenchymal transition (EMT), a process characterized by loss of intercellular adhesion properties and acquire of mesenchymal-like phenotype in epithelial cells, is associated with tumor invasion, migration, and metastasis (Pastushenko and Blanpain 2019). Ferroptosis, an iron-dependent form of cell death triggered by excessive lipid peroxidation, has been confirmed in playing an important role in the development and therapy of tumors (Hassannia et al., 2019). Ferroptosis reagents such as erastin and RSL3 could induce ferroptosis and suppress tumor development (Chen et al., 2021). Intriguingly, it is reported that EMT signaling can increase the cellular sensitivity to ferroptosis in tumors (Chen et al., 2021). For example, transcription factor ZEB1 can stimulate EMT-mediated tumor metastasis and high transcript levels of ZEB1 can sensitize cancer cells to ferroptosis through promoting the increase of PPAR*γ*, a major regulator of lipid metabolism in liver (Viswanathan et al., 2017). Cancer cells with resistance to traditional treatment or high metastasis tendency may be particularly prone to ferroptosis, thus opening up a new field of targeted therapy research. EMT signals can also promote ferroptosis in human cancer cell lines and organs, the high degree of interstitial cell status is associated with the susceptibility to ferroptosis (Hanahan and Weinberg 2011; Chen et al., 2021). Therefore, we speculated that the application of ferroptosis inducer in tumors with EMT may be a potential therapeutic strategy to suppress tumor progress.

Our goal is to provide insight and summary on the mechanism and function of ferroptosis in tumor development and its potential therapeutic targets. The aim of our study is finding novel prognostic biomarkers for identification of in COAD patients for ferroptosis-related therapy. In this study, the mRNA expression data and clinical information of COAD patients from The Cancer Genome Atlas (TCGA) datasets were used to screen some genes related to metastasis and ferroptosis. Membrane fatty acid composition has attracted more and more attention in promoting cell survival, lipotoxicity limitation and ferroptosis. In addition, it is now clear that tumor cells show plasticity in fatty acid metabolism and respond to extratumoral and systemic metabolic signals (such as obesity and tumor treatment) to promote the development of aggressive, treatment resistant and related diseases (Hoy et al., 2021). A patients’ BMI level in COAD datasets was a critical strategy for screening. Then, we established a four-gene prognostic signature based on these screened genes. The effectiveness of the prognostic model was verified using another mRNA data from Gene Expression Omnibus (GEO) datasets. Moreover, a nomogram was constructed to predict overall survival and confirmed the prognostic value of our prediction model. Additionally, weighted gene co-expression network analysis (WGCNA) further demonstrated that two of the four genes are closely correlative with liver and lymphatic invasion of COAD. Our four-gene signature may provide new insights for the survival predictions and treatment options of COAD.

## Materials and Methods

### Microarray Data Collection and Preprocessing

521 The level-3 mRNA primary expression data and corresponding phenotype data of 521 primary COAD samples were downloaded from the TCGA database (Astolfi et al., 2020), then were collected and preprocessed. Of these, 521 samples contained prognostic information. The gene expression data were log2-transformed (normalized RSEM count +1). Genes w *i*th low or no expression were then picked out from the analysis. The standardized mRNA data of GSE75500 (Evert van den Broek et al., 2016 Nov 8) were obtained from GEO database (https://www.ncbi.nlm.nih.gov/geo/). Specifically, these datasets included 114 COAD samples processed on the GPL16694 Agilent-022522 SurePrint G3 CGH Array 4x180K (Probe Name Version) of GSE75500, which were applied as validation sets. Quality controls included relative expression (RLE) and standardized scale-free standard error (NUSE) implemented in the affyPLM package provided by Bioconductor (Wang J et al., 2021) (www.bioconductor.org). Raw gene expression data were background corrected using the Robust Multi-Array Average (RMA) method, standardized using the Quantiles method, and summarized by the median polish method. The above methods were all well applied in the flexible package provided from Bioconductor. According to the annotation file in the platform provided by the chip manufacturing (GPL16694 for GSE75500), the probe label is converted into a genetic symbol. The average expression values were set as relative expression values of genes which were matched with multiple probes. Additionally, 28 proliferation and metastasis regulatory genes were identified from the human molecular characterization database V7.2 (MSIgDB). 521 patients with primary COAD in TCGA were selected as the experimental group. The characteristic genes were screened and the TNM staging model was established. To identify differentially expressed genes in samples from advanced patients (stage Ⅲ and stage IV), a Bayes test was performed (FDR<0.05). The above differentially expressed genes were intersected with 28 proliferative and metastatic genes, and the proliferation and metastasis related genes correlated with the prognosis of COAD were preliminarily obtained.

### Differentially Expressed Genes Screening

The differentially expressed genes (DEGs) of the TCGA clinical staging dataset were screened by limma (Ritchie et al., 2015) package, and the results were visualized by volcano plot. The volcano plot was generated by ggplot2 (Ito and Murphy 2013). |Log2FC| > 1, *p* < 0.05 were set as the cutoffs for the DEGs.

### Functional Analysis

Gene ontology (GO) (The Gene Ontology 2017) and Kyoto Encyclopedia of Genes and Genomes (KEGG) (Kanehisa et al., 2017)pathway enrichment analysis were performed to identify the functions of DEGs using the cluster Profile package. GO enrichment analysis included cellular component (CC), molecular function (MF), and biological process (BP), and the parameters were set to *p* < 0.01, minimum count >3, enrichment factor >1.5. *p* < 0.05 was considered statistically significant. The Gene Set Enrichment Analysis (GSEA) (Aravind Subramanian et al., 2005) was used to perform pathway enrichment for DEGs, and 2000 alignments per analysis. The KEGG pathways data set in the curated gene sets was selected. The threshold for the statistically significant GSEA analysis was set to the corrected *p* < 0.05 and false discovery rate (FDR) < 0.25. Selecting “c2.cp.kegg.v7.0.symbols.gmt” as a reference gene set. *p* < 0.05 and FDR<0.25 was considered to be significantly enriched. The result of enrichment analysis would be characterized by corrected *p* values and NES. GSEA enrichment analysis and visualization were performed using GSEA local software.

### Determination of Final Prognostic Signature Using Lasso Cox Regression Analysis

Lasso Cox regression analysis was used to determine the final prognostic signature using glmnet (Jerome Friedman and Tibshirani, 2010). Genes identified in the univariate analysis as covariates were included in the multivariate Cox regression analysis to determine their effect on the OS. The prognostic model was established by combining the expression values of the selected genes with multivariate Cox regression coefficients, that is, the risk score of each patient was equal to the sum of the gene expression values and multivariate Cox regression coefficients. The median risk score was selected as the threshold to stratify COAD patients for the evaluation of the model prediction effect. Patients with the risk score greater than the threshold were divided into high-risk group, and the remaining patients to low-risk group. The difference in survival rate between the two groups was evaluated by a log-rank test. The author plotted the survival curve using fit plot.

### Nomogram and Risk Assessment Analysis

Risk score was accessed using time-dependent receiver operating characteristic (ROC) curves to predict specificity and sensitivity of COAD patients’ survival at 1, 3, and 5 years of follow-up, while risk scores and other clinical indicators (age, sex, TNM stage, and histological grade) were observed. The ability of the model to predict OS was verified in the validation datasets (GSE75500). In addition, based on the increased value of risk, a dot plot was used to show the distribution of patient mortality events. Heat map was used to observe the expression distribution of each characteristic gene in two different risk groups. To test the independence and reliability of the prognostic model, we selected clinically common prognostic indicators for COAD (age, sex, TNM stage, and histological grade). Among these possible prognostic factors, risk score and age were used as continuous variables, and gender (male/female), TNM stage (III-IV/I-II), and histological grade (4/3/2/1) were converted into categorical variables. Univariate and multivariate Cox regression analyses were performed for clinical characteristics and risk scores of COAD patients in the TCGA cohort to identify clinical factors associated with survival. Log-rank test was used to verify whether the risk score was related to other survival related clinical information. Variables that could be used as independent prognostic factors were then used to establish a nomogram.

### PPI Network Construction

String Database (http://string-db.org, version 11) (Szklarczyk et al., 2021) was used to construct protein-protein interaction networks. All the related differential molecules after the intersection of different mRNAs were put into the database for analysis, and the interaction threshold was set at 0.4. Cytoscape software was used to visualize molecular interaction networks. A plug-in of Cytoscape software: Cytohubba was used to analyze hub genes in the network.

### Receiver Operating Characteristic Diagnostic Prediction of Genes and Risk Assessment

ROC prediction analysis was performed to acquire the specificity and sensitivity of each gene on the screening significant key gene using time ROC package (Blanche et al., 2013). Survival package was used to calculate the clinical parameters and grouping parameters for Survival prognosis and cumulative risk factor.

### Weighted Gene Co-expression Network Analysis (WGCNA)

WGCNA was conducted using the WGCNA package (Guy et al., 2019). It is a systematic way for effectively acquiring the expression patterns of multiple genes in different samples, which can obtain a gene cluster with the same clinical phenotype.

### Statistical Analysis

All statistical analysis considered the calculation of mean and standard deviation, and was performed using R software 3.4.0.3. *p* < 0.05 was considered statistically significant. Univariate and multivariate Cox regressions were analyzed by COXPH function. Lasso-Cox regression analysis was conducted with R software package glmnet. In addition, the Log-rank test was performed using the survdiff (Tan et al., 2020) function in the survival package. The heatmap of characteristic gene expression was drawn by ggplot2 and heatmap function. The R software package rms (Zhang et al., 2019) was used to achieve the establishment and application of the nomogram. The calibration analysis (Guy et al., 2019) was performed aligned with nomogram. Decision curve analysis (DCA) (Van Calster et al., 2018) was conducted to verify the risk model.

## Results

### Screening of Ferroptosis-Related Differentially Expressed Genes

**FIGURE 1:**
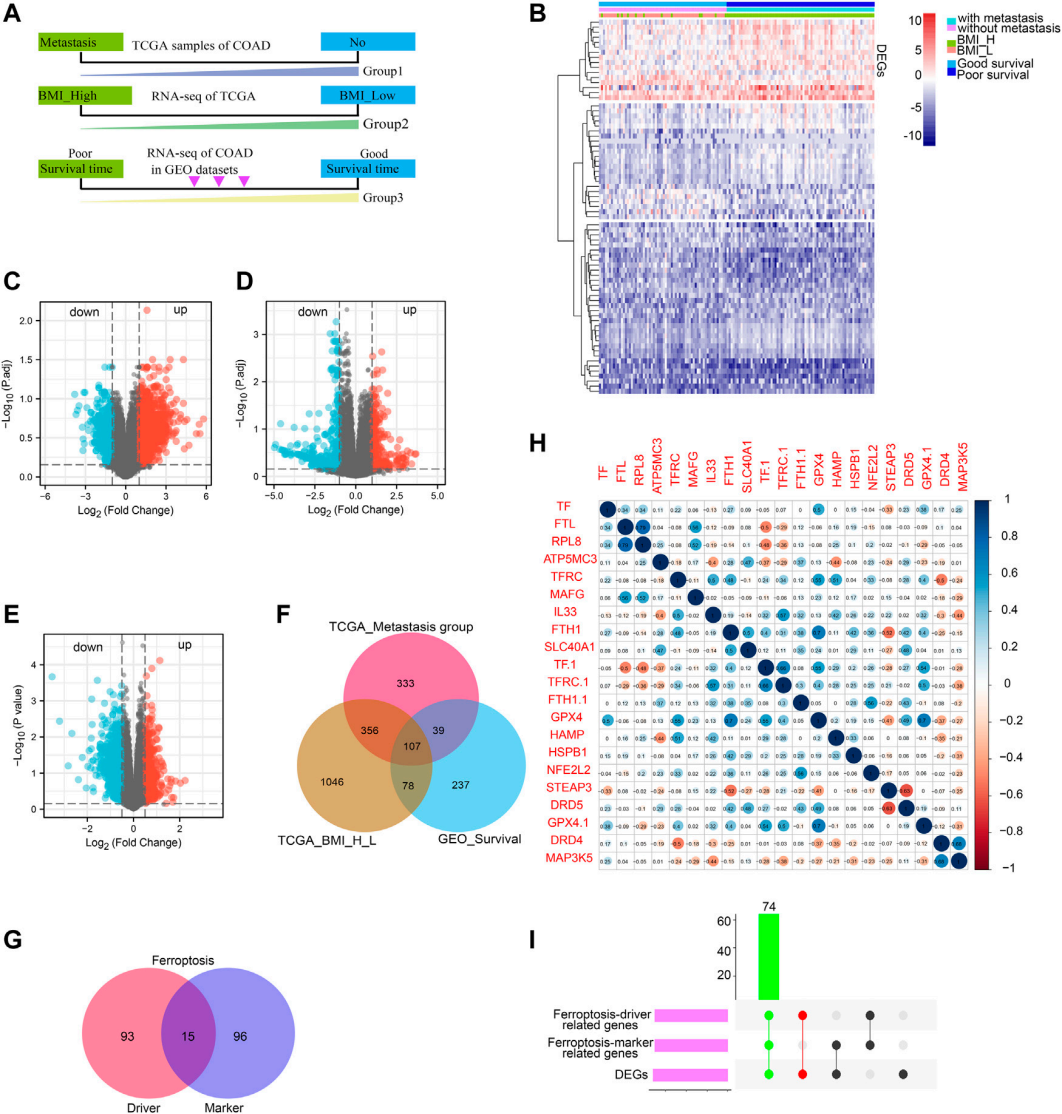
Screening of ferroptosis-related DEGs. Work flow of screening based on TCGA COAD metastasis, BMI value and survival status of GEO COAD patients, respectively **(A)**; heat map of DEGs based on TCGA COAD metastasis, BMI value and survival status of GEO COAD patients **(B)**, different bars indicated different group; Volcanic map of DEGs, red represents upregulated genes while blue represents downregulated genes **(C–E)**; Venn diagram showing upregulated overlapping DEGs based on TCGA COAD metastasis, BMI value and survival status of GEO COAD patients, respectively **(F)**. Ferroptosis-driver and -marker genes were shown with venn diagram from FerrDb database **(G)**; Ferroptosis-driver genes were 108; Ferroptosis-marker genes were 111; the intersected genes co-expressed between ferroptosis-driver and -marker genes were 15; The correlation between DEGs and ferroptosis-related genes was shown with representative correlation plot **(H)**; Blue bar indicated: R (sperman’s coefficient) > 0; red bar indicated: R (sperman’s coefficient) < 0; The DEGs correlated positively with ferroptosis-driver and -marker genes were presented with UpsetR diagram **(I)**; green bar indicated the final ferroptosis-related genes.

The COAD patients in TCGA datasets were divided into two groups by metastasis status (with metastasis and without metastasis) and BMI relative value (BMI_H, BMI_L). Besides, The COAD patients in GEO datasets were also divided into two groups according to the patient’s survival status (good survival time and poor survival time) (Figure 1A). Then, we identified the DEGs by comparing the RNA-expression of the three groups of patients in the TCGA and GEO datasets. The DEGs were displayed in the heat map (Figure 1B). The volcano plot of the up-regulated DEGs and down-regulated DEGs (*p* < 0.05) was shown in Figures 1C–E. According to the clinical stage, 875 up-regulated genes were obtained in metastasis group. In addition, there were 1527 up-regulated genes in BMI_H group in the TCGA dataset, and 401 up-regulated genes by patient survival status in the GEO dataset. A Venn diagram displayed 107 DEGs obtained through the intersection of the above up-regulated DEGs (Figure 1F). And then we downloaded ferroptosis Driver and Marker genes from the FerrDb database, the numbers of ferroptosis Driver and Marker genes were shown with venn diagram (Figure 1G). Correlation analysis was conducted between 107 DEGs and ferroptosis Driver and Marker genes, and the representative results were displayed in a correlation plot (Figure 1H). Finally, the 74 intersected genes tightly correlated with ferroptosis were identified (Figure 1I).

### Functional Enrichment Analysis of Ferroptosis Related Differentially Expressed Genes and Screening of Epithelial-to-Mesenchymal Transition Related Genes by Gene Set Enrichment Analysis

**FIGURE 2:**
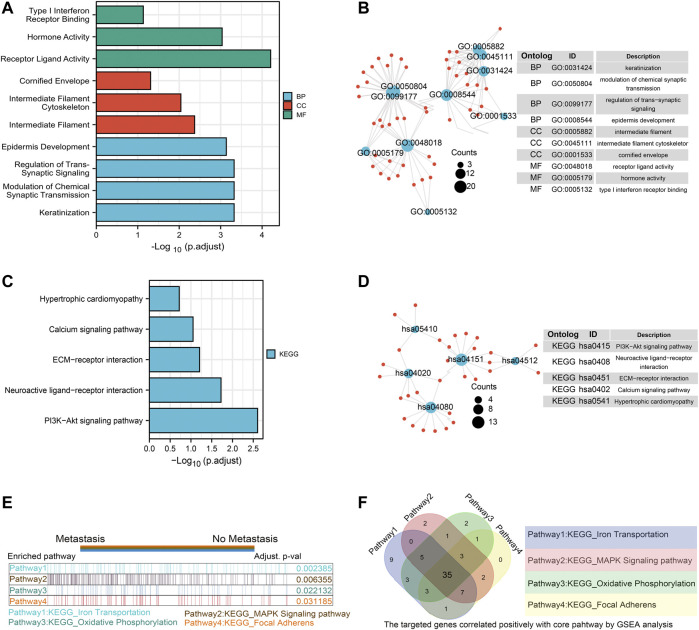
Functional enrichment analysis of ferroptosis related DEGs and Screening of EMT related genes by GSEA. GO enrichment analysis of DEGs. Blue represents Biological Process, red represents Cellular Component, and green represents Molecular Function **(A,B)**; KEGG enrichment analysis of DEGs. Color gradient represents *p* value. The size of the circle represents the number of enrichment **(C,D)**; The gene set enrichment analysis (GSEA) was conducted between “metastasis” group and “without metastasis” group, the core pathways were selected, *p* value < 0.05, FDR<0.25 **(** **E** **)**; the correlation analysis was performed between the 74 DEGS and gene sets from four core pathways, the results were shown with venn diagram **(** **F** **)**.

Furthermore, we conducted GO/KEGG pathway enrichment analysis on these intersected DEGs. For BP, DEGs were significantly enriched in typeⅠinterferon receptor binding, receptor ligand activity, hormone activity and other pathways related to interferon, hormone synthesis pathway and receptor coupling pathway. The CCs analysis indicated that DEGs were mainly enriched in intermediate filament cytoskeleton, intermediate filament and other cellular components associated with intracellular fibrous skeleton protein remodeling and connection. In MF, DEGs mainly enriched in the regulation of *trans*-synaptic signaling, modulation of chemical synaptic transmission and other key functions of intercellular signaling transmission correlated to synaptic signal transmission, remodeling and regulation (Figures 2A,B). Key information was shown in Table 1. The KEGG pathway analysis revealed that DEGs were mostly enriched in pathways in focal Adhesion, hippo signaling pathway, MAPK signaling pathway and other pivotal biological signaling pathways such as invasion, migration, and intercellular junctions, as well as PI3K-Akt signaling pathway, calcium signaling pathway and other signaling pathways related to carcinogenic and calcium channels regulation (Figures 2C,D). The key enrichment information was sorted into Table2. And then The TCGA COAD samples were divided into the “Metastasis” group and the “No Metastasis” group by metastasis status. Positive correlation enrichment pathways were acquired by GSEA, mainly including Focal adherens, Iron transportation, MAPK signaling pathway, oxidative phosphorylation and other pathways related to invasion, migration, carcinogenesis in BP(Figure 2E). Furthermore, we extract a gene set of some critical metastasis-related signaling pathways, including Focal adherens, iron transportation, MAPK signaling pathway, oxidative phosphorylation. Correlation analysis was conducted between 74 DEGs screened above and the genes tightly correlated with metastasis, and the results were displayed in a venn diagram (Figure 2F). Finally, we identified 35 genes that are highly associated with the core signaling pathways for further model screening.

**TABLE 1:** GO enrichment analysis of DEGs.

Ontology	ID	Description	Gene ratio	Bg ratio	*p* value	p.adjust	*q* value
BP	GO: 0031424	Keratinization	13/190	224/18,670	5.16e-07	4.73e-04	4.26e-04
BP	GO: 0050804	Modulation of chemical synaptic transmission	18/190	436/18,670	5.36e-07	4.73e-04	4.26e-04
BP	GO: 0099177	Regulation of *trans*-synaptic signaling	18/190	437/18,670	5.54e-07	4.73e-04	4.26e-04
BP	GO: 0008544	*Epidermis* development	18/190	464/18,670	1.31e-06	7.31e-04	6.58e-04
BP	GO: 0043588	Skin development	17/190	419/18,670	1.43e-06	7.31e-04	6.58e-04
CC	GO: 0005882	Intermediate filament	11/208	214/19,717	1.78e-05	0.004	0.004
CC	GO: 0045111	Intermediate filament cytoskeleton	11/208	251/19,717	7.62e-05	0.009	0.008
CC	GO: 0001533	Cornified envelope	5/208	65/19,717	6.15e-04	0.049	0.043
MF	GO: 0048018	Receptor ligand activity	20/187	482/17,697	2.00e-07	6.22e-05	5.85e-05
MF	GO: 0005179	Hormone activity	9/187	122/17,697	5.92e-06	9.21e-04	8.66e-04
MF	GO: 0005132	Type I interferon receptor binding	3/187	17/17,697	7.08e-04	0.073	0.069

**TABLE 2:** KEGG enrichment analysis of DEGs.

Ontology	ID	Description	Gene ratio	Bg ratio	*p* value	p. adjust	*q* value
KEGG	hsa04145	Phagosome	13/159	152/8076	8.42e-06	0.002	0.002
KEGG	hsa04512	ECM-receptor interaction	7/159	88/8076	0.002	0.141	0.138
KEGG	hsa04514	Cell adhesion molecules	9/159	149/8076	0.003	0.141	0.138
KEGG	hsa05144	Malaria	5/159	50/8076	0.003	0.141	0.138
KEGG	hsa04390	Hippo signaling pathway	9/159	157/8076	0.004	0.141	0.138

### Lasso-Cox Analysis and Screening of Key Target Genes

**FIGURE 3:**
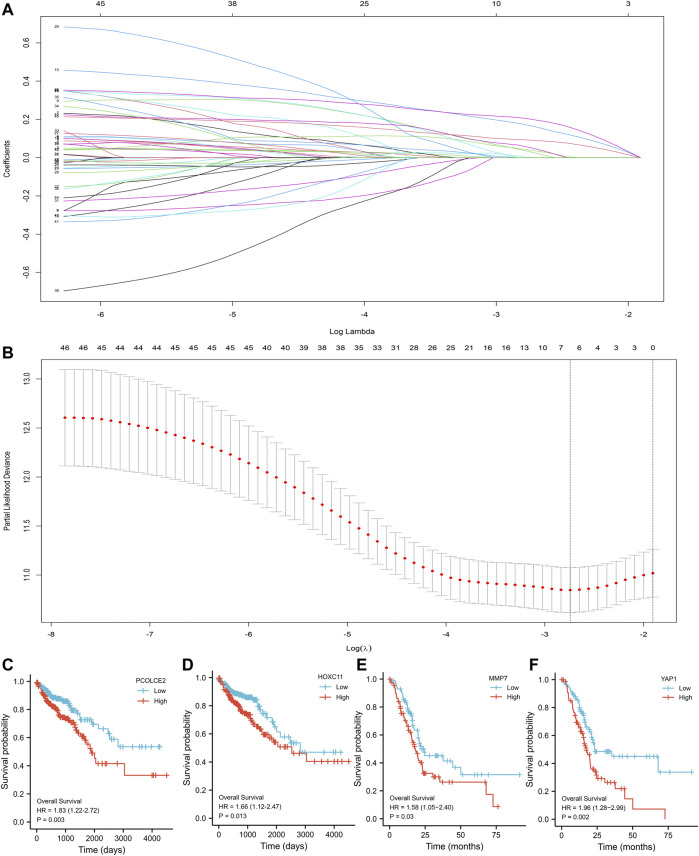
Screening of key variables by Lasso-Cox regression. Screening of the key variables associated with invasion and metastasis by Lasso-Cox regression **(A)**; Variable analysis by Lasso regression correction **(B)**; Kaplan–Meier survival analysis of COAD patients with high and low expression of PCOLCE2 **(C)**, HOXC11 **(D)**, MMP7 **(E)**, YAP1 **(F)**. *p* value was statistically significant, HR > 1.5.

**FIGURE 4:**
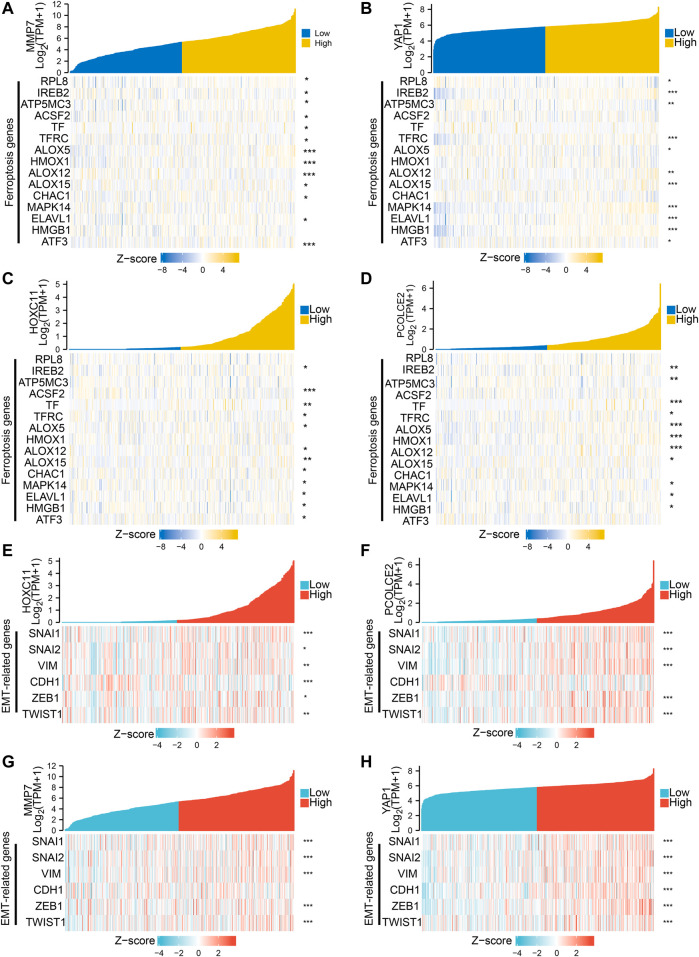
Screening of genes associated with both ferroptosis and invasive migration. The correlation between MMP7 **(A)**, YAP1 **(B)**, HOXC11 **(C)**, PCOLCE2 **(D)**, and ferroptosis genes was conducted and presented with Single gene co-expression heat map; The correlation between HOXC11 **(E)**,PCOLCE2 **(F)**,MMP7 **(G)**, YAP1 **(H)** and EMT-related genes was performed and shown with Single gene co-expression heat map.

The Lasso regression analysis was performed and four genes were identified (PCOLCE2, MMP7, YAP1, and HOXC11) (Figures 3A,B). To determine the role of these four genes in COAD prognosis, Kaplan–Meier survival analysis was used to compare patient survival prognosis between the high expression and low expression groups of each prognostic gene. The results showed that the survival rate of patients in the high expression group was much lower than that in the low expression group (*p* < 0.05, HR > 1.5). Compared with low expression group of PCOLCE2, the median survival time in high expression group was significantly smaller (HR = 1.83, *p* = 0.003) (Figure 3C). Likewise, the median survival time in high expression group of HOXC11 was also significantly smaller than in lower expression group (H = 1.66, *p* = 0.013) (Figure 3D), and MMP7, YAP1 presented the same results (MMP7, H = 1.58, *p* = 0.03; YAP1, H = 1.96, *p* = 0.002) (Figures 3E,F). And, the tight correlation between MMP7 (Figure 4A), YAP1 (Figure 4B), HOXC11 (Figure 4C), PCOLCE2 (Figure 4D) and ferroptosis genes were shown with co-expressed heatmap for validating efficiency of screening. Besides, the correlation analysis between HOXC11 (Figure 4E), PCOLCE2 (Figure 4F), MMP7 (Figure 4G), YAP1 (Figure 4H) and EMT-related genes were also performed for further exploring targeted genes; the results indicated that these four genes were tightly correlated with both ferroptosis and EMT process.

### Establishment of a Risk Prediction Model Based on Invasion and Transfer Genes

**FIGURE 5:**
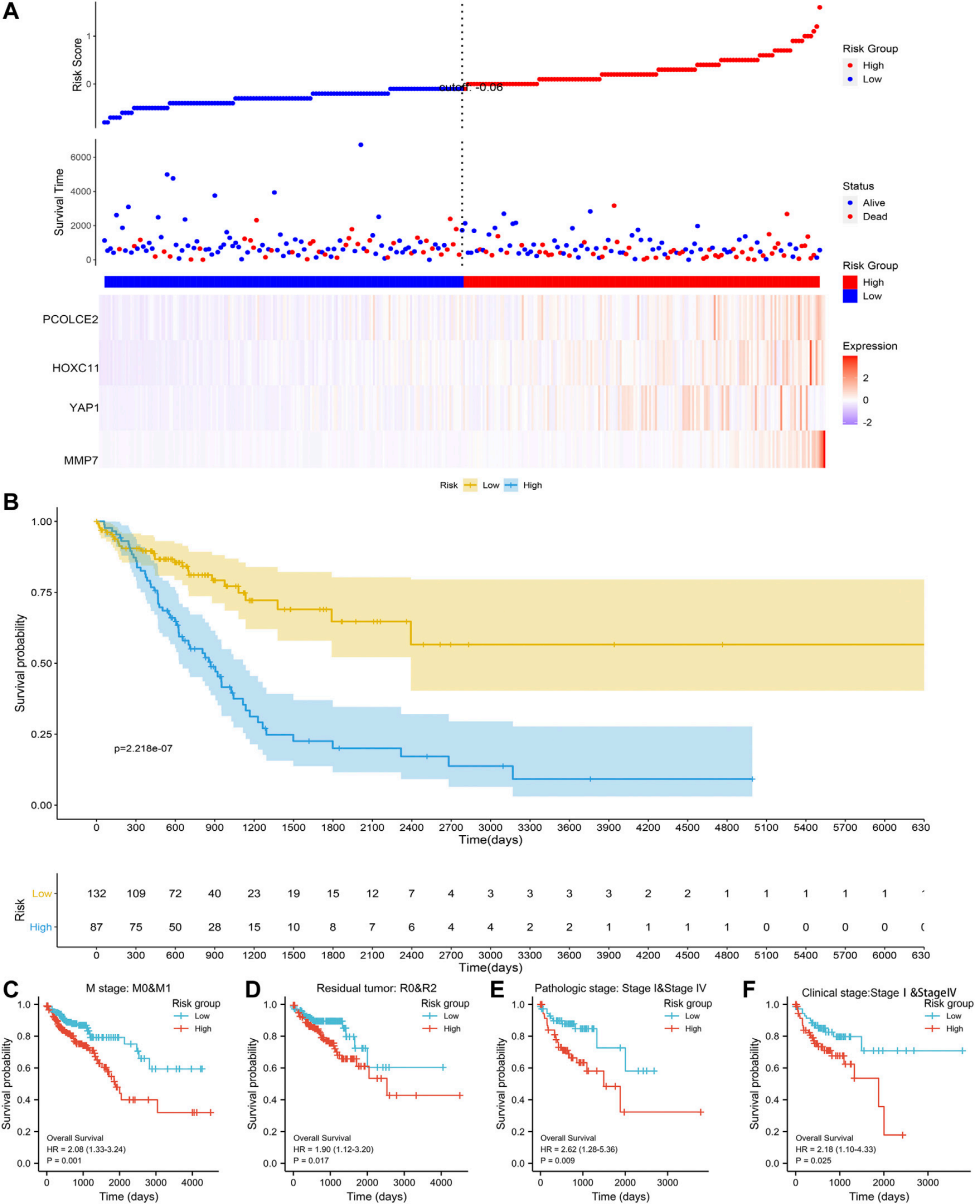
Establishment of the risk signature model. The establishment of the risk signaturemodel based on PCOLCE2, YAP1, MMP7, and HOXC11 gene in the TCGA **(A)**; Comparison of clinical prognosis between high and low risk groups divided using the median risk score in the TCGA **(B)**; Comparison of survival prognosis between high and low risk groups by COAD distant metastasis **(C)**, tumor recurrence **(D)**, clinical stage **(E)**, and pathological grade **(F)**, respectively, p values were statistically significant.

Based on the above four prognostic genes (PCOLCE2, MMP7, YAP1 and HOXC11), we constructed a metastasis-related prognostic model. In our model, the higher the risk score, the earlier the patient event occurred, and the higher the expression of the four genes (Figure 5A). Multivariate regression analysis was performed for each prognostic gene to obtain the regression coefficient of each gene. 521 COAD samples were evaluated using TCGA cohort, and each sample was given a risk score and assigned to a risk group. Firstly, to evaluate the effectiveness of the model, we compared the survival differences between the high risk and low risk groups. Results showed that when the four screened predicted genes were highly expressed, the OS of the patients was significantly decreased, and the prognosis of high risk group was significantly worse than that of the low risk group (*p* < 0.0001) (Figure 5B). To further evaluate the predictive performance of this risk model, different clinical parameters between high and low risk groups were selected for model validation. Results showed that the median OS of high risk group is significantly shorter than that of low risk group, regardless of in M stage (M0&M1), tumor recurrence (R0&R2), histomathological grade (Ⅰ&Ⅳ) and clinical stages (Ⅰ&Ⅳ) (Figures 5C–F). The above results showed that we have established an effective prognostic model for COAD associated with proliferation and metastasis.

To exclude the possibility of model overfitting in TCGA, we verified the model in GEO dataset. We analyzed the prognosis and the expression level of the four genes between high risk and low risk group in the GEO dataset. Results showed that the four prognostic genes expression is higher in high risk group, and the prognosis is worse in high risk group than that in low risk group (Supplementary Figure S1A). The median OS of patients in high risk group was significantly (*p* < 0.0001) shorter than that in low-risk group. In addition, we also verified the effectiveness of the model by combining with clinical characteristics and risk score with nomogram model and calibration analysis in 1, 5, 10 survival years (Supplementary Figures S1B,C). The median OS of high risk group was significantly shorter than that of low risk group, regardless of in tumor recurrence (R0&R2), T stage (T1&T3) and lymphatic invasion (transfer & no transfer) (Supplementary Figures S1D–F). The results indicated that this model related to proliferation and metastasis was robust on different platforms.

### Identification of Key Prognostic Genes Particularly Containing PCOLCE2/HOXC11

**FIGURE 6:**
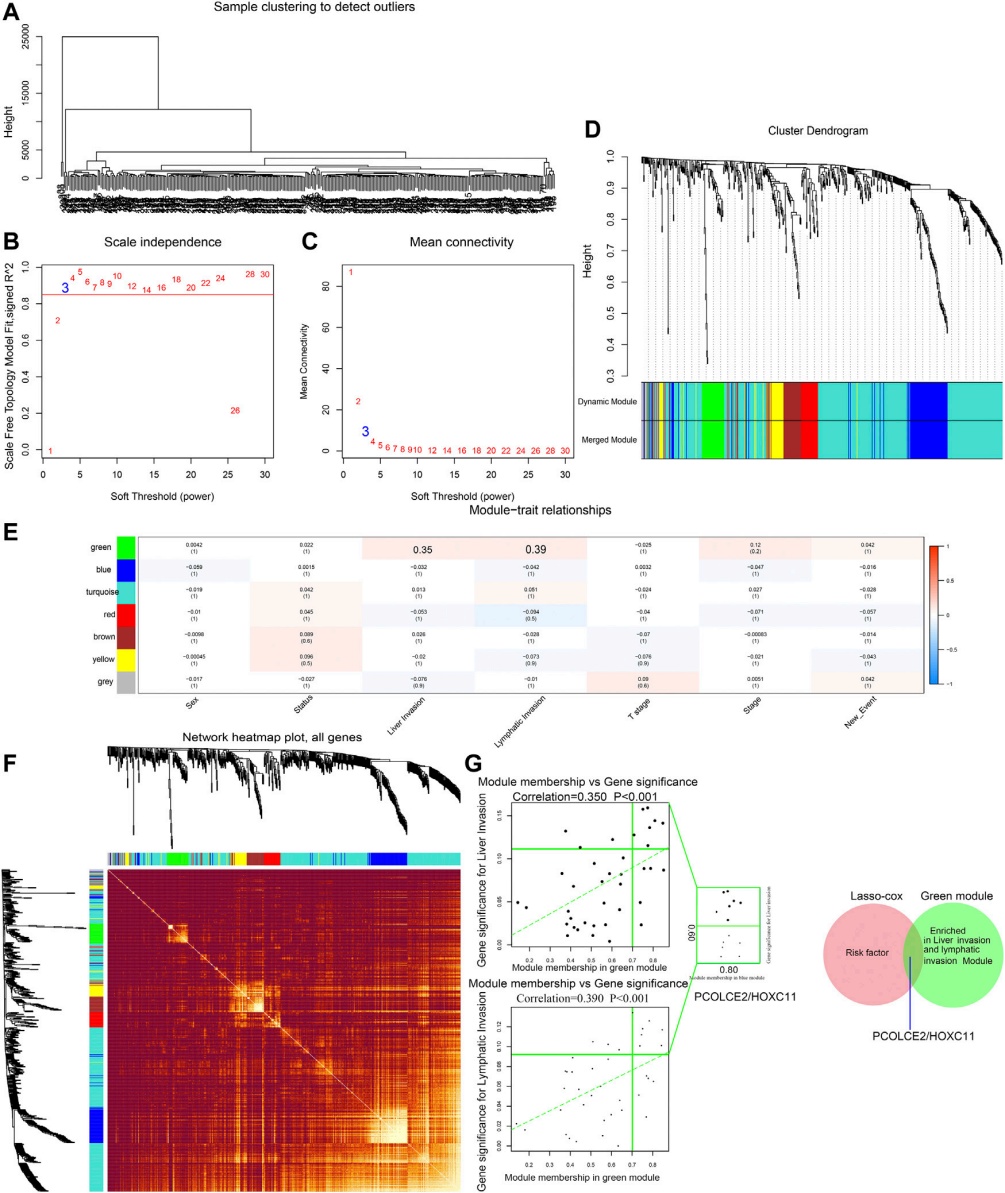
Screening of key phenotypic module and key genes by WGCNA. Hierarchical clustering dendrogram of samples from TCGA database **(A)**. Analysis of scale-free fit index and the mean connectivity for various soft-thresholding powers. Testing the scale-free topology when *β* = 3 **(B)**. Hierarchical clustering dendrogram of genes with dissimilarity on the basis of topological overlap. Modules are the branches of the clustering tree **(C)**. Correlation between module genes and clinical traits. Each row corresponds to module genes, and columns represent clinical traits. Each cell contains the correlation and *p* value, and the green module containing PCOLCE2/HOXC11 are selected. The red bar represents positive correlation, while the blue bar represents negative correlation **(D)**. The heat map describes the TOM among the 1000 genes selected in WGCNA. Gene modules are represented by horizontal and vertical coordinates, and highly overlapping modules are marked by high-bright parts **(E)**. The green module containing PCOLCE2/HOXC11 was correlated with COAD liver and lymphatic invasion. A scatter plot was mapped between GS and MM (green module and liver invasion) in COAD patients **(F)**. The intersected gene, PCOLCE2/HOXC11, was identified between risk factors in the risk model, and genes contained in green modules with Venn diagram **(G)**.

**FIGURE 7:**
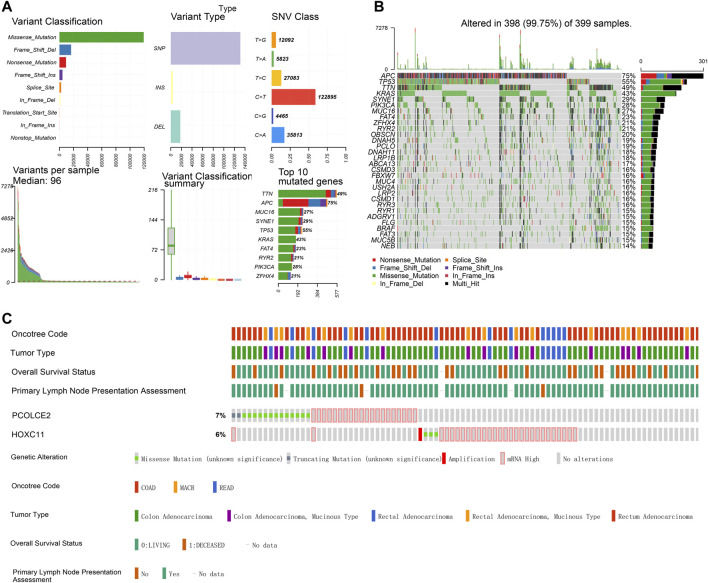
Single-nucleotide variation (SNV) and copy number variation (CNV) of targeted genes in TCGA samples. Percentage and types of SNV of COAD samples in TCGA datasets **(A)**. Top ranking genes with high percentage of SNV in high risk score group of COAD samples in TCGA datasets **(B)**. The percentage of CNV of PCOLCE2, HOXC11 in COAD samples of TCGA **(C)**.

WGCNA is a systematic way for effectively acquiring the expression patterns of multiple genes in different samples, which can obtain a gene cluster with the same expression pattern. The association between modules and phenotype of samples such as clinical traits can be studied. A total of 234 samples with clinical characteristics were included in WGCNA (Figure 6A). In this study, the power of *β* = 3 (scale-free R2 = 0.850) was selected as the soft threshold to establish the scale-free network (Figures 6B,C). Therefore, six co-expressed modules were identified after removing the gray modules by combined dynamic tree cutting (Figure 6D). The TOM was mapped to 1000 genes selected in the analysis, indicating that each module was independently verified. We found that the green module containing PCOLCE2/HOXC11 was correlated with COAD liver invasion and lymphatic invasion (R2 >0.3, *p* < 0.05). A scatter plot was mapped between GS and MM (green module and liver, lymphatic invasion) in COAD patients. Therefore, PCOLCE2/HOXC11 was considered to be one of the critical genes in the green module. PCOLCE2/HOXC11 was identified as the common target gene between the risk model and weighted co-expressed network through Venn diagram (Figures 6E–G). The percentage and types of SNV of COAD in TCGA samples were presented (Figure 7A), and top ranking genes with high percentage of SNV in high risk score group of COAD samples were shown (Figure 7B). The results indicated that high risk score group accompanied higher percentage of SNV which predicted poor prognosis. Besides, the percentage of copy number variation (PCOLCE2, 7%; HOXC11, 6%) was calculated in TCGA samples (Figure 7C). Furthermore, ROC analysis was used to determine the prognostic values of these two genes. The corresponding area under curve (AUC) value for HOXC11 was 0.734 (Supplementary Figure S2A), and the AUC value for PCOLCE2 was 0.950 (Supplementary Figure S2E). Additionally, the expression of the two prognostic genes in COAD was analyzed according to various clinicopathological features, including NM tumor stage, lymph node metastasis and peritoneal metastasis. The results revealed that the expression level of PCOLCE2 and HOXC11 in COAD patients was significantly higher than that in healthy individuals, regardless of for M tumor stage, lymph node metastasis and peritoneal metastasis (*p* < 0.05) (Supplementary Figures S2B–D,F–H). Overall, the results further confirmed that the two genes screened in this study were unfavorable factors for the survival status of COAD patients. In addition, we further analyzed the clinical pathological factors and risk scores (*p* < 0.05, HR > 1) by univariate and multivariate Cox regression analysis, which further showed the prognostic value of the risk model in these four genes. The correlation between the clinical characteristics, the risk score and the expression level of the two genes of COAD patients in the TCGA cohort and the prognosis was verified. Univariate Cox regression analysis showed that risk score and TNM stage were significantly correlated with OS (*p* < 0.0001). Meanwhile, we established a nomogram model using clinicopathological factors, risk scores and these two key genes in combination with 1, 5, and 10 years survival times, confirming that the expression levels of these two genes could more accurately predict 1-, 5- and 10-years survival time (Supplementary Figure S3A), and Time ROC analysis of these two genes (HOCX11 and PCOLCE2) had prediction ability in 1, 3, 5 survival time (AUC>0.5) (Supplementary Figures S3B,C). Additionally, combined with the clinical information of patients, we selected 433 patients in the high risk and low risk groups for univariate and multivariate forest prediction, and found that HOCX11 and PCOLCE2 both had high risk (HR > 1), which has a negative effect on the prognosis of patients (Supplementary Figures 3D,E). And the table was listed for reference (Table3), which further demonstrated the prognostic value of these two genes.

**TABLE 3:** Univariate and multivariate analysis of targeted genes combing with clinical factors.

Characteristics	Total (N)	Univariate analysis		Multivariate analysis	
		Hazard ratio (95% CI)	*p* value	Hazard ratio (95% CI)	*p* value
T stage (T3&T4 vs. T1&T2)	476	3.072 (1.423–6.631)	0.004	3.201 (0.980–10.455)	0.054
N stage (N1&N2 vs. N0)	477	2.592 (1.743–3.855)	<0.001	1.149 (0.643–2.054)	0.639
M stage (M1 vs. M0)	414	4.193 (2.683–6.554)	<0.001	2.537 (1.450–4.436)	0.001
HOXC11 (High vs. Low)	477	1.661 (1.115–2.474)	0.013	1.578 (0.973–2.559)	0.065
PCOLCE2 (High vs. Low)	477	1.826 (1.224–2.725)	0.003	1.640 (1.006–2.674)	0.047

### Validation for the Expression of Predicted Hub Genes

**FIGURE 8:**
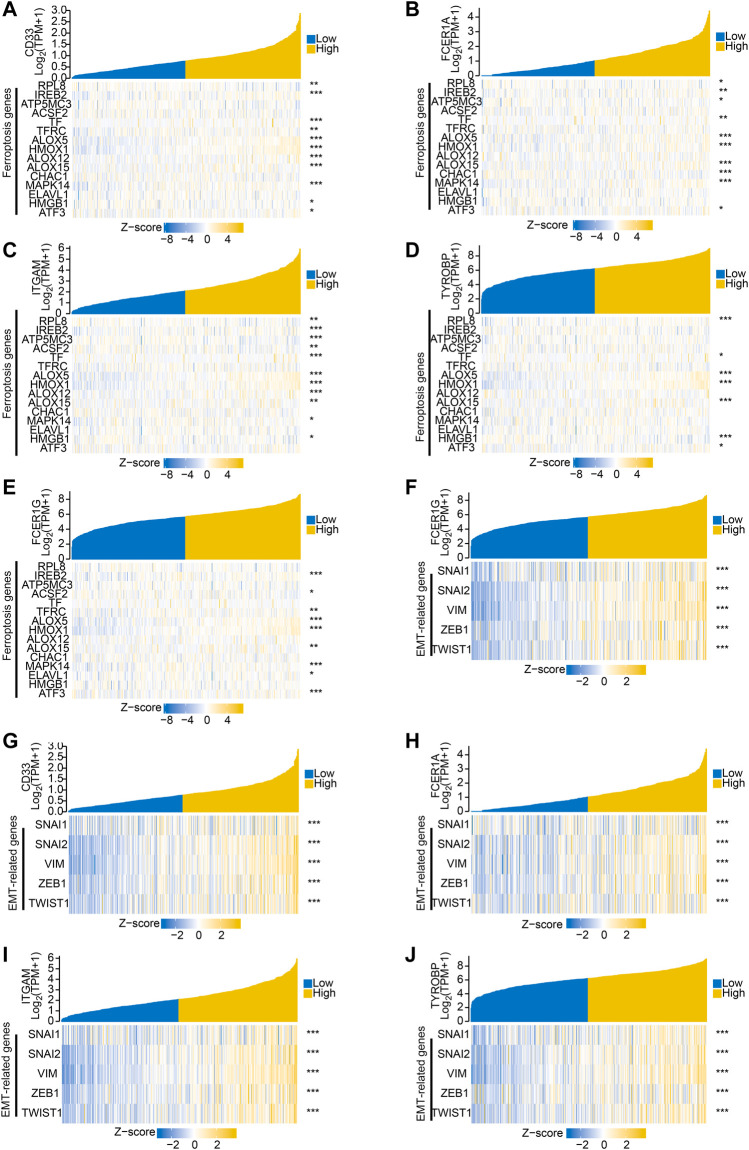
Hub genes associated with both ferroptosis and invasive migration. The correlation between CD33 **(A)**, FCER1A **(B)**, ITGAM **(C)**, TYROBP **(D)**, FCER1G **(E)** and ferroptosis genes was conducted and presented with Single gene co-expression heat map; The correlation between FCER1G **(F)**, CD33 **(G)**, FCER1A **(H)**, ITGAM **(I)**, TYROBP **(J)** and EMT-related genes was performed and shown with Single gene co-expression heat map.

To further explore the related functions of the prognostic genes (PCOLCE2 and HOXC11), we performed a correlation analysis. The positively correlated genes of PCOLCE2 and HOXC11 were respectively identified and showed by a heat map (Supplementary Figures S4A,B). All the acquired genes were performed GO/KEGG pathway enrichment analysis. Results showed that the positively correlative genes were mainly enriched in the cargo receptor activity, scavenger receptor activity and amyloid-beta binding pathway in BP. For CC, the positively correlative genes are significantly enriched in neuron to neuron synapse, axon part, collagene-containing extracellular matrix regulation. The MF analysis revealed that these genes mostly enriched in the regulation of cation channel activity, extracellular structure organization, axonogenesis and other pathways related to key intercellular signal transduction, including extracellular matrix remodeling and signal pathway regulation (Supplementary Figure S4C); However, KEGG enrichment pathways were mostly enriched in hippo signaling pathway, cell adhesion molecules and ECM-receptor interaction and other key biological signaling pathways related to migration, invasion and tight intercellular junctions (Supplementary Figure S4D). Finally, the detailed information of the key enrichment information was sorted into Table4. Subsequently, we performed protein-protein interaction analysis on these related genes to explore their interactions and connections. Finally, the top 10 key target genes in all related genes were obtained using cytohubba, including ITGAM, FCER1G, CD33, SNAP25, ITGAX, LAIR1, TYROBP, FCGR1A and ODLR1 (Supplementary Figures S4E,F). And, the tight correlation between CD33 (Figure 8A), FCER1A (Figure 8B), ITGAM(Figure 8C), TYROBP(Figure 8D), FCER1G (Figure 8E) and ferroptosis genes were shown with co-expressed heatmap for validating efficiency of screening. Besides, the correlation analysis between FCER1G (Figure 8F), CD33 (Figure 8G), FCER1A (Figure 8H), ITGAM(Figure 8I), TYROBP(Figure 8J) and EMT-related genes were also performed for further exploring targeted genes; the results indicated that these hub genes were tightly correlated with both ferroptosis and EMT process. Immunohistochemistry of the nine genes (ITGAM, FCER1G, CD33, SNAP25, ITGAX, LAIR1, TYROBP, FCGR1A, ODLR1) in tumor and normal colon tissues were shown in Supplementary Figures S5A–H. The protein expression levels of these hub genes were significantly up-regulated in tumor tissues compared with normal tissues. The results may explain the low survival rate in the high risk group at the molecular level, indicating that the risk model we established is reliable.

**TABLE 4:** GO enrichment analysis of positively correlated genes.

Ontology	ID	Description	Gene ratio	Bg ratio	*p* value	p.adjust	*q* value
BP	GO: 0007409	Axonogenesis	30/347	468/18,670	4.15e-09	1.59e-05	1.30e-05
BP	GO: 0043062	Extracellular structure organization	25/347	422/18,670	3.80e-07	7.26e-04	5.94e-04
BP	GO: 2001257	Regulation of cation channel activity	15/347	178/18,670	1.24e-06	0.001	9.03e-04
BP	GO: 0030282	Bone mineralization	12/347	114/18,670	1.42e-06	0.001	9.03e-04
BP	GO: 0010959	Regulation of metal ion transport	23/347	394/18,670	1.44e-06	0.001	9.03e-04
CC	GO: 0062023	Collagen-containing extracellular matrix	27/364	406/19,717	1.06e-08	4.02e-06	2.78e-06
CC	GO: 0033267	Axon part	24/364	382/19,717	2.04e-07	3.87e-05	2.67e-05
CC	GO: 0098984	Neuron to neuron synapse	22/364	350/19,717	6.57e-07	8.30e-05	5.74e-05
CC	GO: 0042383	Sarcolemma	13/364	136/19,717	1.46e-06	1.39e-04	9.59e-05
CC	GO: 0097060	Synaptic membrane	24/364	432/19,717	1.84e-06	1.39e-04	9.65e-05
MF	GO: 0005539	Glycosaminoglycan binding	21/343	229/17,697	4.17e-09	2.25e-06	1.97e-06
MF	GO: 0001540	Amyloid-beta binding	12/343	78/17,697	3.20e-08	8.62e-06	7.56e-06
MF	GO: 0005044	Scavenger receptor activity	9/343	51/17,697	5.16e-07	9.28e-05	8.13e-05
MF	GO: 0038024	Cargo receptor activity	11/343	85/17,697	7.33e-07	9.88e-05	8.66e-05
MF	GO: 0008201	Heparin binding	15/343	169/17,697	1.07e-06	1.16e-04	1.02e-04

## Discussion

COAD is a highly malignant cancer with poor prognosis. Although some clinical predictors such as age, gender and TNM stage could predict the prognosis of COAD, the predictive capacity and suitable individualized treatment still needs further improvement due to the high heterogeneity of this cancer. Thus, it is necessary to find novel prognostic biomarkers for COAD. In the present study, we established and validated a four-gene signature which can effectively predict the survival rate of patients with COAD. Moreover, this prognostic risk model may provide potential biomarkers for identification of COAD patients suitable for ferroptosis-induced therapy.

In our study, a total 107 overlapping DEGs were obtained by integrative analysis of multiple datasets. Then, GO/KEGG pathway enrichment analysis were conducted and revealed that the screened DEGs are associated with pathways of tumor invasion and migration, including focal adhesion and MAPK signaling pathway. 35 DEGs were generated through the intersection of the above 107 DEGs related to iron transportation, MAPK signaling pathway, oxidative phosphorylation and focal adherence. Furthermore, four genes were screened by Lasso regression analysis and used to construct a four-gene signature as a prognostic risk model. Moreover, the robustness and reliability of this model were validated in GEO dataset. Subsequently, we employed WGCNA analysis and found that PCOLCE2 and HOXC11 are involved in liver and lymphatic invasion of COAD. Ten positively correlated genes of PCOLCE2 and HOXC11 were identified through correlation analysis and protein-protein interaction analysis. Additionally, we demonstrated that the protein expression levels of these hub genes were significantly up-regulated in tumor tissues compared with normal samples, which further insuring the reliability of our prognostic model.

MMP7, a member of the matrix metalloproteinase family, has an ability to degrade the extracellular matrix (Winer et al., 2018). MMP7 participates in the development of multiple malignant tumors and is associated with the invasion and metastasis of cancers, including CRC (Gao et al., 2019), lung cancer (Stenvold et al., 2012), pancreatic cancer (Fukuda et al., 2011; Chen et al., 2013), tongue squamous cell carcinoma (Yuan et al., 2020) and prostate cancer (Lynch et al., 2005). A previous study showed that Yes-associated protein 1 (YAP1) contributed to the invasion and migration of non-small cell lung cancer by promoting Slug transcription via the transcription co-factor TEAD (Yu et al., 2018). High YAP1 levels suggested low overall survival rates in CRC and are one of the potential prognostic biomarkers for CRC (Zihui Xu et al., 2019). Interestingly, the activation of transcription factor YAP1 could promote the susceptibility of tumor cells to ferroptosis by controlling the expression of ferroptosis modulatory factors such as TFRC, EMP1, ACSL4, and ANGPTL4 (Viswanathan et al., 2017; Wu et al., 2019), which suggests that ferroptosis-inducing drugs may be a perfect treatment for YAP1-activated tumors. PCOLCE2 is a gene encoding procollagen C-endopeptidase enhancer protein 2 (PCPE2) (Xu et al., 2000). PCPE2, a 52-kDa glycoprotein, is located in the extracellular matrix and can stimulate bone morphogenetic protein 1 (BMP1) activity on fibrillar procollagens (Moali et al., 2005). Interestingly, it has been demonstrated that BMP1 is involved in the metastasis of gastric cancer and non-small cell lung cancer (Wu et al., 2014; Hsieh et al., 2018). Additionally, it is indicated that PCOLCE (procollagen C-proteinase enhancer protein) acts a pivotal part in promoting the lung metastasis of osteosarcoma (Wang et al., 2019). A previous study demonstrated that PCOLCE performed well in predicting overall survivals of colorectal cancer patients (Chen et al., 2019). Thus, combining with our results, we believe that PCOLCE2 has a close relationship with EMT-related metastasis of COAD. Homeobox C11 gene (HOXC11), one member of the HOX family, was shown to be closely related to the clinical outcome of patients with renal cancer, cervical cancer or breast cancer (McIlroy et al., 2010; Liu et al., 2015; Eoh et al., 2017). Moreover, overexpressing HOXC11 was found to promote cancer cell proliferation and migration (Liu et al., 2015; Peng et al., 2021). In summary, the four prognostic genes we screened were associated with cancer development and metastasis, thereby could predict the prognosis of COAD.

At this time, ferroptosis has attracted increased attention in the mechanism of tumor development. There is accumulating evidence indicating that induction of ferroptosis might be an effective therapeutic strategy to prevent tumor resistance and suppress tumor growth (Roh et al., 2016; Hangauer et al., 2017; Viswanathan et al., 2017; Tsoi et al., 2018). Additionally, some cancer cells with mesenchymal-like phenotype are sensitive to ferroptosis (Chen et al., 2021). These findings suggest that using ferroptosis-inducing drugs may become a special and significant therapeutic method for tumors expressing certain factors involved in EMT.

In this study, we screened four genes related to EMT and ferroptosis and established a reliable, stable and effective four-gene risk prediction model in COAD patients. High metastasis tendency may be particularly prone to ferroptosis, thus opening up a new field of targeted therapy research. Our goal is to provide insight and summary on ferroptosis-related genes signature in tumor invasion and metastasis and its potential therapeutic targets. This signature may perform well as a sign to identify some COAD patients suitable with ferroptosis induction therapy. Nevertheless, there are some limitations in our study. Selection bias may generate due to the limited sample size. For better clinical application, further studies using larger sample quantity are necessary to ensure the reliability of our finding. Moreover, clinical samples and animal experiments for comprehensive verification are needed to understand the potential molecular mechanism of this type of COAD.

## Conclusion

In this study, we established a four-gene prognostic signature (MMP7, YAP1, PCOLCE and HOXC11) based on EMT and ferroptosis related genes and validated the reliability and effectiveness of this model in COAD. Moreover, through WGCNA analysis, we found that PCOLCE and HOXC11 were correlative with liver and lymphatic invasion in patients with COAD. These four genes may become potential prognostic biomarkers to identify COAD with metastasis. Moreover, this four-gene signature may be able to determine the COAD suitable with ferroptosis induction therapy. Overall, this signature provided novel possibilities for better prognostic evaluation of COAD patients and would be of great guiding significance for individualized treatment and clinical decision.

## Data Availability

The original contributions presented in the study are publicly available. This data can be found here: https://github.com/Xieyongjie666/Code-availability/commit/8de1946ea8b980ced4288d0114135386cb0dbcc3.

## Supplementary Material

Supplementary Figure S1 Verification of risk signature model The risk signature model based on PCOLCE2, YAP1, MMP7 and HOXC11 gene was verified in GEO COAD datasets with survival analysis **(A)**. Nomogram was presented with risk scores and other clinical factors **(B)**. Calibration analysis was conducted with risk scores in 1, 3, 5 year **(C)**. Comparison of survival prognosis between high and low risk groups by lymphatic metastasis of COAD **(D)**, tumor recurrence **(E)**, clinical stage **(F)**, respectively, P values were statistically significant.

Supplementary Figure S2 The basic characteristics of invasion and migration related gene: PCOLCE2, HOXC11 ROC analysis of PCOLCE2 **(A)** and HOXC11 **(E)** indicates the diagnosis and prediction of COAD; Boxplots showing the relative expression of PCOLCE2 **(B,D)** in COAD patients by lymph node metastasis, T and M tumor stage, and peritoneal metastasis, respectively; Boxplots showing the relative expression of HOXC11 **(F,H)** in COAD patients by lymph node metastasis, T and M tumor stage and peritoneal metastasis, respectively.

Supplementary Figure S3 Prediction ability of two intersected genes: PCOLCE2, HOXC11 The establishment of nomogram model with clinical factors, and expression of PCOLCE2, HOXC11 **(A)**. Time ROC analysis of PCOLCE2, HOXC11 in 1, 3, 5 year survival time **(B,C)**. univariate and multivariate cox analysis of the potential targeted genes, PCOLCE2, HOXC11 **(D,E)**.

Supplementary Figure S4 Functional enrichment analysis of positively correlated genes of targeted genes Heat map of positive correlated gene of PCOLCE2 **(A)** and HOXC11 **(B)**; Go enrichment analysis of positively correlated genes **(C)**, including Biological Process (BP) analysis, Cellular Components (CC) analysis, Molecular Function (MF) analysis; Visualization for KEGG pathway enrichment analysis of positively correlated genes **(D)**; PPI network interaction analysis of positively correlated genes **(E)**; Screening of hub gene **(F)**.

Supplementary Figure S5 Representative immunohistochemistry staining of predicted hub gene The protein expression levels of PCOLCE2 **(A)**, ITGAM **(B)**, TYROBP **(C)**, CD33 **(D)**, HOXC11 **(E)**, ITGAX **(F)**, FCER1A **(G)**, FCER1G **(H)** in normal individuals or COAD patients.
