# Supplementary figures and images for: Identification of Ferroptosis-Related Genes Signature Predicting the Efficiency of Invasion and Metastasis Ability in Colon Adenocarcinoma
Source: Front Cell Dev Biol. 2022 Jan 26;9:815104. doi: 10.3389/fcell.2021.815104 (PMC8826729; doi:10.3389/fcell.2021.815104)

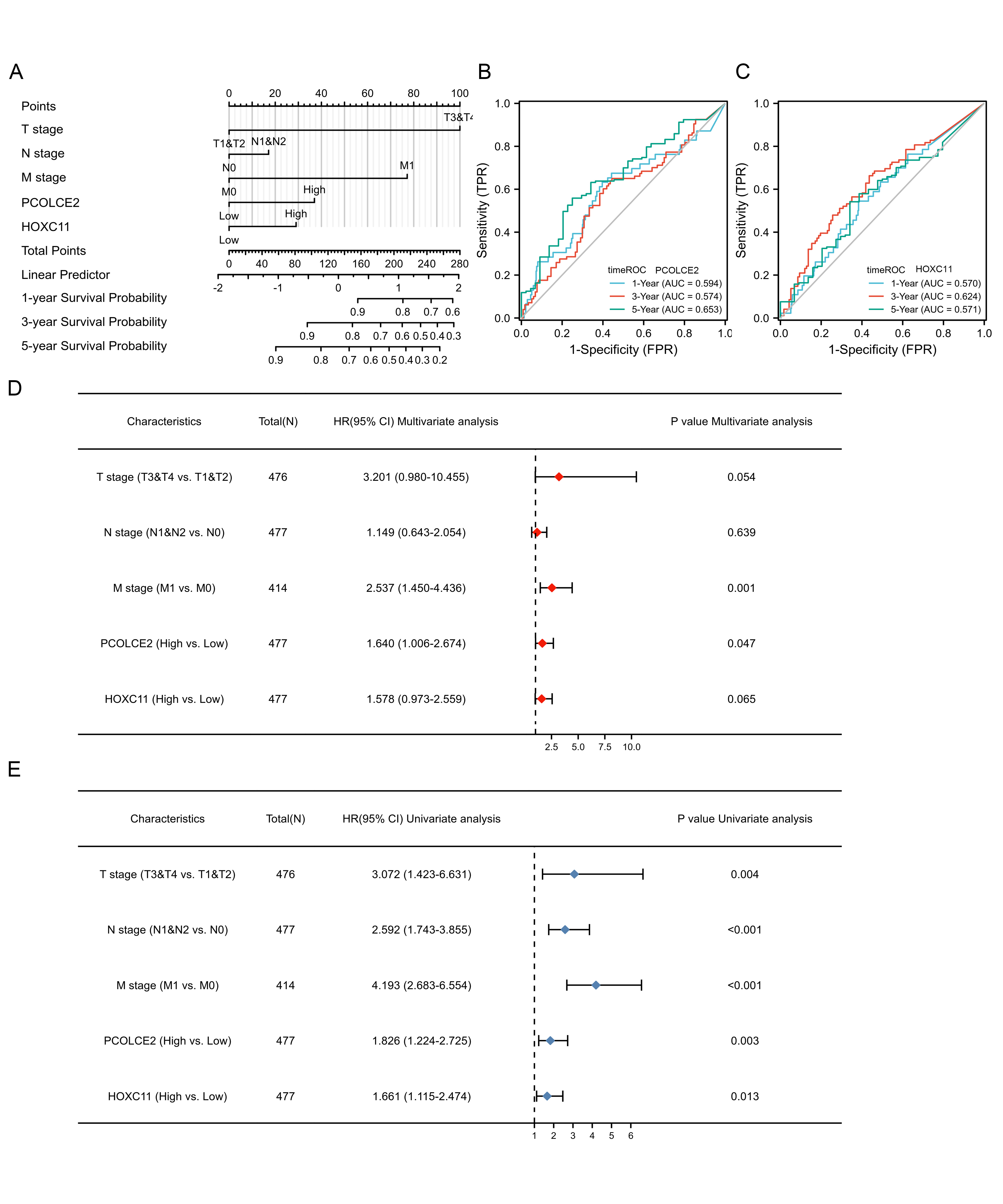

Supplement: Supplementary file 1 [file Image3.JPEG]

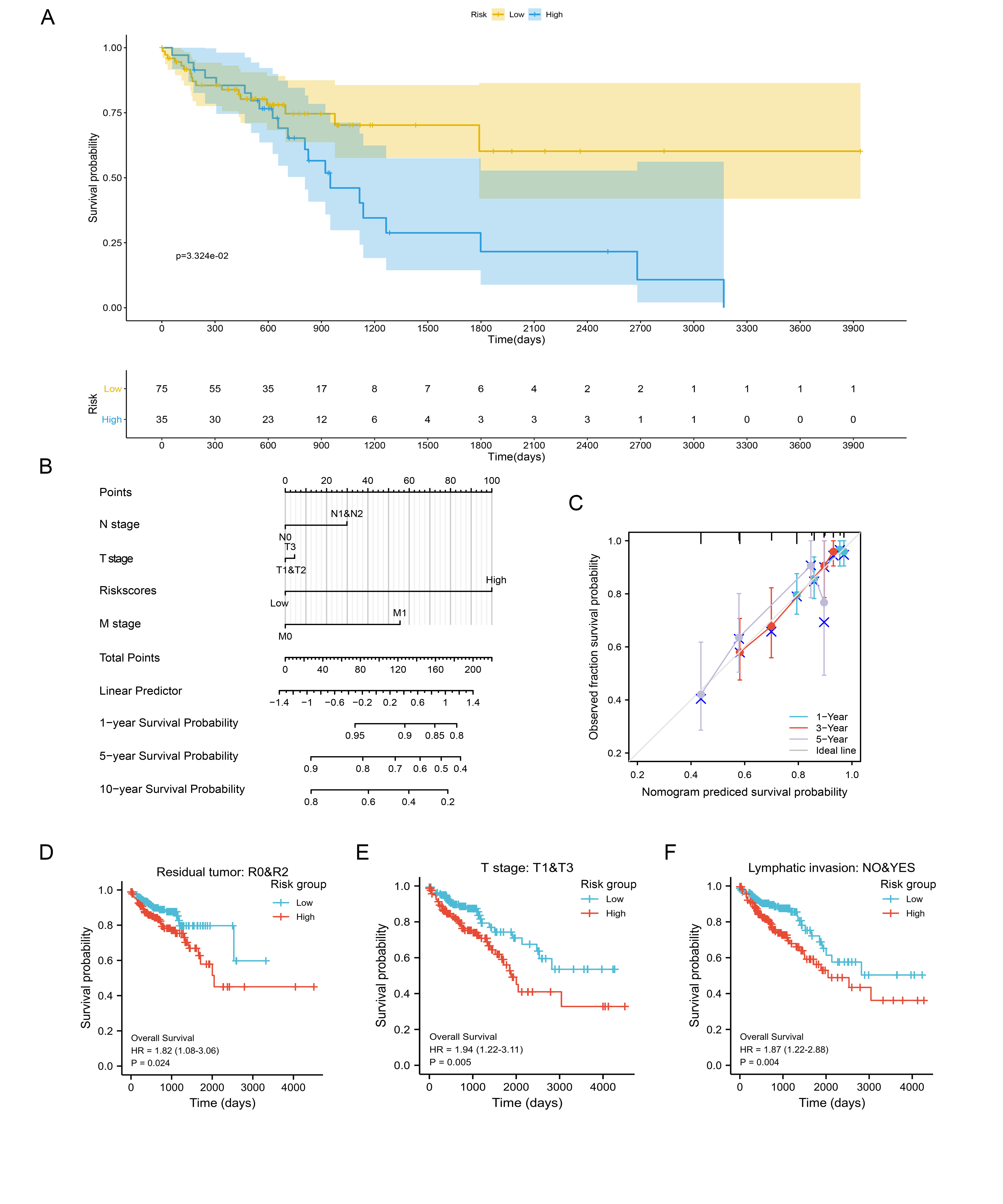

Supplement: Supplementary file 2 [file Image1.JPEG]

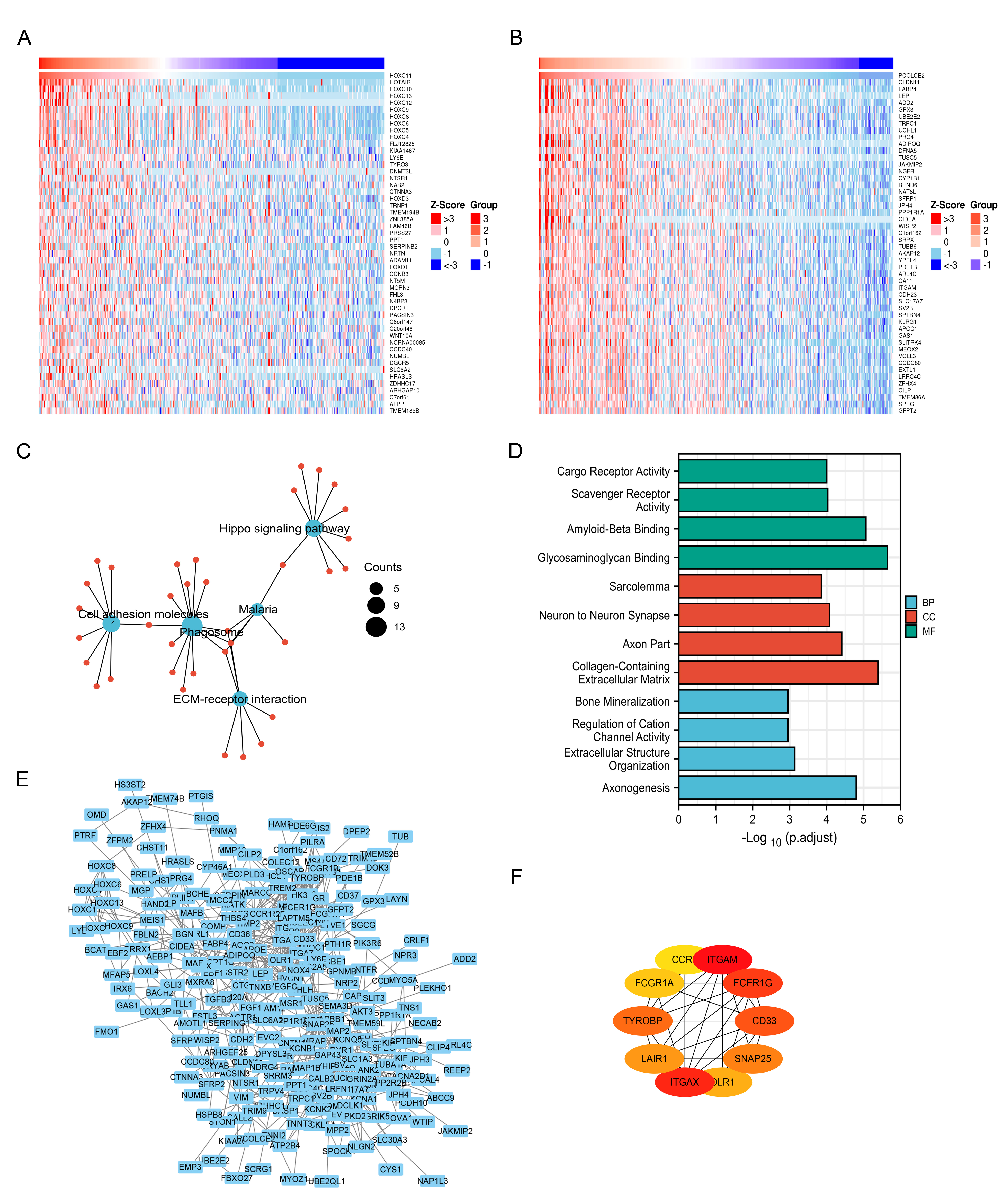

Supplement: Supplementary file 3 [file Image4.JPEG]

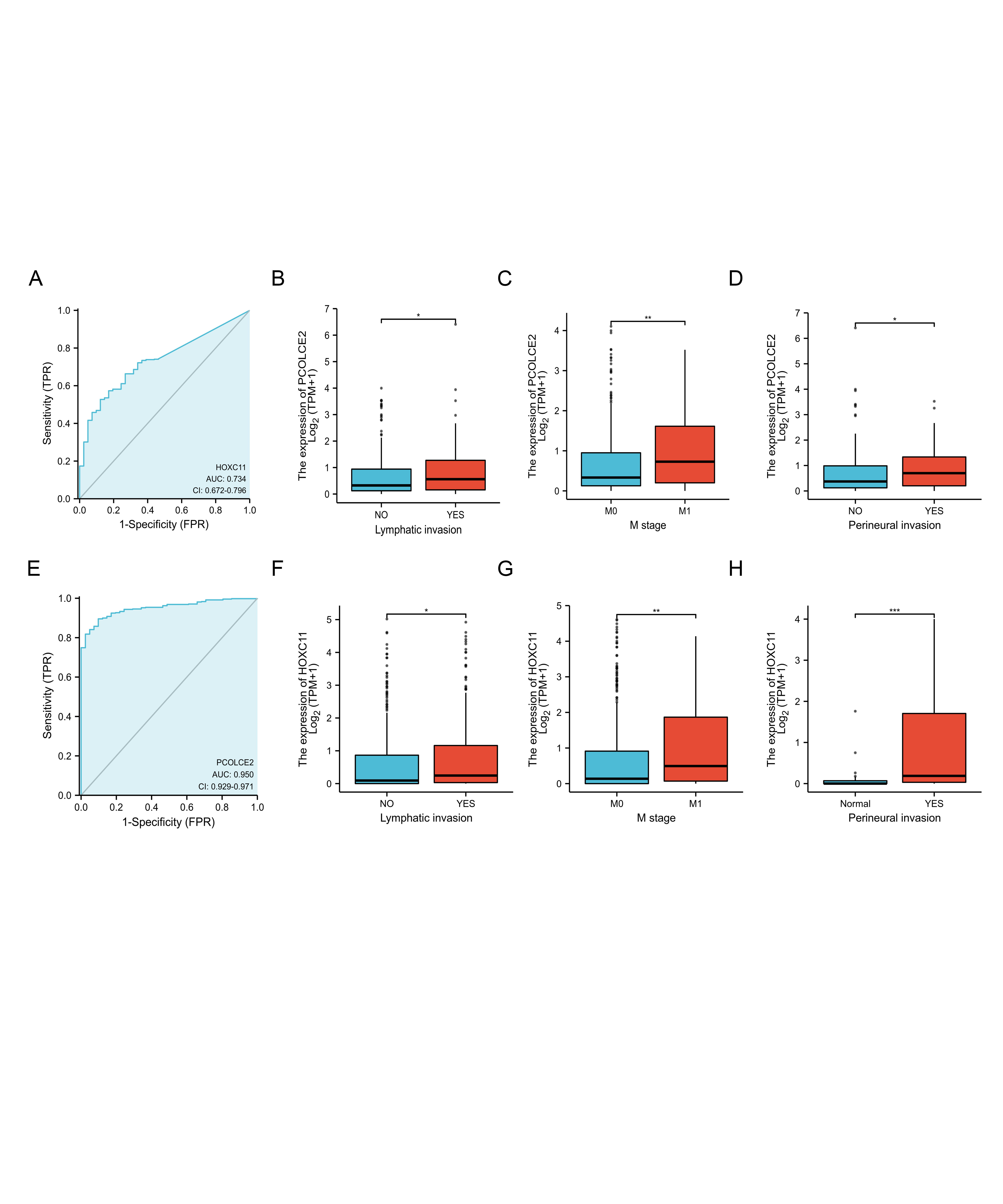

Supplement: Supplementary file 4 [file Image2.JPEG]
